# Regulation of AMPA receptors in spinal nociception

**DOI:** 10.1186/1744-8069-6-5

**Published:** 2010-01-21

**Authors:** Yun Wang, Jing Wu, Zhiguo Wu, Qing Lin, Yun Yue, Li Fang

**Affiliations:** 1 Department of Anesthesiology, Beijing Chaoyang Hospital, Capital Medical University, Beijing 100020, PR China; 2 Department of Anesthesiology and Critical Care Medicine, Johns Hopkins University School of Medicine, Baltimore, MD 21205, USA; 3 Department of Neuro-Oncology, The University of Texas MD Anderson Cancer Center, Houston, TX 77030, USA; 4 Division of Neurosurgery, Department of Surgery, The University of Texas Medical Branch, Galveston, TX 77555-0517, USA; 5 Department of Neuroscience and Cell Biology, The University of Texas Medical Branch, Galveston, TX 77555-0517, USA

## Abstract

The functional properties of α-amino-3-hydroxy-5-methy-4-isoxazole propionate (AMPA) receptors in different brain regions, such as hippocampus and cerebellum, have been well studied *in vitro *and *in vivo*. The AMPA receptors present a unique characteristic in the mechanisms of subunit regulation during LTP (long-term potentiation) and LTD (long-term depression), which are involved in the trafficking, altered composition and phosphorylation of AMPA receptor subunits. Accumulated data have demonstrated that spinal AMPA receptors play a critical role in the mechanism of both acute and persistent pain. However, less is known about the biochemical regulation of AMPA receptor subunits in the spinal cord in response to painful stimuli. Recent studies have shown that some important regulatory processes, such as the trafficking of AMPA receptor subunit, subunit compositional changes, phosphorylation of AMPA receptor subunits, and their interaction with partner proteins may contribute to spinal nociceptive transmission. Of all these regulation processes, the phosphorylation of AMPA receptor subunits is the most important since it may trigger or affect other cellular processes. Therefore, these study results may suggest an effective strategy in developing novel analgesics targeting AMPA receptor subunit regulation that may be useful in treating persistent and chronic pain without unacceptable side effects in the clinics.

## Introduction

Glutamate synapses are involved in most excitatory neurotransmission in the central nervous system (CNS). The major glutamate receptor subtypes at glutamatergic synapses are currently subdivided into ionotropic glutamate receptors (ion channel forming) and metabotropic glutamate receptors (G-protein coupled). The former may include N-methyl-D-aspartate (NMDA) receptors and non-NMDA receptors, such as AMPA and kainite receptors. Cumulative evidence suggests that activity-dependent changes in the efficacy of glutamatergic synapses in pain transmission pathways greatly contribute to chronic pain caused by tissue damage or nerve injuries [1,2]. A great number of studies have addressed the role of NMDA receptors and metabotropic glutamate receptors in synapses between primary afferent fibers and spinal neurons. It has been demonstrated that the activation of NMDA receptors and metabotropic glutamate receptors critically contributed to the development of chronic nociceptive hypersensitivity following peripheral tissue damage or nerve injuries [1]. In contrast, the AMPA glutamate receptors are originally thought to mediate rapid excitatory neurotransmission in the CNS. Recently, more studies had supported the critical contributions of spinal AMPA receptors in the development of both acute and chronic painful responses [3-6].

AMPA receptors are widely distributed in the CNS. The functional properties and regulations of AMPA receptors in different brain regions, such as in the hippocampus (during long-term potentiation) and the cerebellum (during long-term depression), have been well studied both *in vitro *and *in vivo*. These studies suggest that the glutamate-mediated excitatory synaptic transmission efficiency is dependent on the number and function of AMPA receptors at glutamatergic synapses. The former is associated with the trafficking of AMPA receptors and the latter, is influenced by AMPA receptor subunit composition, post-transcriptional and post-translational modifications, and their interacting proteins [7-9]. Interestingly, several studies have shown that the trafficking of AMPA receptors, their subunits composition, phosphorylation regulation of AMPA receptor subunits, and interacting proteins also play an important role in the nociceptive processing of the spinal cord. This review will bring together recent advances in understanding the molecular mechanisms of spinal AMPA receptor regulation and their implications in nociception.

## Structure of AMPA receptor channels, their expression and localization in the spinal cord

As one of three classes of ionotropic glutamate receptors, AMPA receptors were cloned and expressed in recombinant systems in the late 1980s. Four homologous subunits, GluR1 to GluR4, assemble in various combinations to form distinct AMPA receptors. Each subunit includes an N-terminal extracellular amino domain, a ligand-binding domain, a receptor-channel domain, and an intracellular C-terminal domain [10,11]. Two polypeptide segments (S1 and S2) are localized in the ligand-binding domain, which appear to represent the agonist-recognition site. The receptor channel domain consists of three transmembrane domains (M1, M3, and M4) and one re-entrant loop within the membrane (M2). The M2 loop participates in the formation of the ion-channel pore. The C-terminal intracellular regulation domain of theses subunits plays an especially important role in the regulation of receptor function by presenting multiple protein phosphorylation sites for various known protein kinases, such as calcium/calmodulin protein kinases II (CaMKII), protein kinase C (PKC) and protein kinase A (PKA). AMPA receptors are heteromeric molecules comprising various combinations of GluR1, GluR2, GluR3, and GluR4 subunits. In the CNS, GluR1 and GluR2 subunits are ubiquitously expressed and are present in most AMPA receptors in the adult mammalian CNS. In contrast to the GluR1, GluR3 and GluR4 subunits, GluR2 contains an arginine at a critical position in the pore-forming M2 segment. Incorporation of GluR2 into heteromeric AMPA receptor strongly reduces the permeability of influxed Ca^2+ ^ions and modifies current rectification and macroscopic-channel conductance [12].

Immunohistochemical and *in situ *hybridization studies indicate that GluR1-4 subunits are all expressed in the spinal dorsal horn [13]. There is a strong expression of GluR1 in superficial dorsal horn such as in laminae I -II, and a weaker expression in deeper dorsal horn laminae. The expression of GluR2 was observed throughout the dorsal horn and was abundant in inner lamina II and sparse in outer lamina II. The deep laminae, III-IV, show scattered cells staining for GluR1, GluR2/3, and GluR4 [14,15]. Todd's group showed that synaptic AMPA receptors on the dendrites of the lamina III, IV and the NK1 receptor projection neurons contained GluR2, GluR3 and GluR4, but not GluR1 subunits [16]. Since GluR2 is widely expressed in the CNS, a majority of AMPA receptors in the CNS present low permeability for Ca^2+ ^influx. However, a high density of Ca^2+^-permeable AMPA receptors is observed in the post-natal spinal dorsal horn, particularly in the superficial spinal laminae I and II, which may be involved in nociception [17]. Activation of Ca^2+^-permeable AMPA receptors in the spinal dorsal horn can enhance the AMPA receptor-mediated synaptic transmission [17].

## Regulation of the spinal AMPA receptors in post-synaptic membrane through the receptor trafficking evoked by painful stimuli

**Figure 1:**
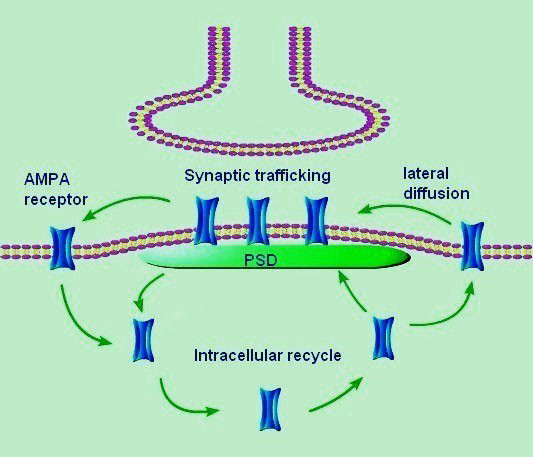
**Pathways of AMPA receptor trafficking**. AMPA receptors may cycle between intracellular stores and the cell surface rapidly and constitutively. AMPA receptors in plasma membrane may exchange between the extra-synaptic and synaptic membrane in a manner of lateral diffusion. The receptor-cycling event and lateral diffusion can regulate the number of AMPA receptors in synapses and further changes the synapse strength. PSD, post-synaptic density.

The trafficking of AMPA receptors has been well studied in glutamatergic neurons of hippocampus. These studies have shown that AMPA receptors present some distinctive characteristics in receptor trafficking from cytosol to postsynaptic membrane (Figure 1). On one hand, AMPA receptors may rapidly and constitutively cycle between intracellular stores and the cellular membrane surface [18,19]. On the other hand, AMPA receptors in plasma membrane may exchange between the extra-synaptic and synaptic membrane in a manner of lateral diffusion [20]. The receptor-cycling event and lateral diffusion can influence the number of AMPA receptors in synapses and further changes the synapse strength. It has been demonstrated that the regulation of AMPA receptor cycling and surface trafficking plays a critical role in the induction of LTP in hippocampal neurons [21]. In contrast to the wealth of information on the regulation of the AMPA receptor trafficking in hippocampal neurons, much less is known about its trafficking events in spinal nociceptive neurons following painful stimuli. Many previous studies suggest that spinal cord central sensitization resembles LTP in the hippocampus [22,23]. Part of the mechanisms underlying the LTP in the hippocampus may also apply to the spinal cord. The current evidence supports the involvement of AMPA receptor trafficking in spinal nociception. In a mice model of visceral hyperalgesia, Galan et al. demonstrated that a significant trafficking event of GluR1 subunit of AMPA receptors from cytosol to plasma membrane in spinal neurons existed and was evoked by painful visceral stimuli [24]. Peripheral injury may alter spinal AMPA receptor composition through the transcriptional regulation of subunit gene expression in an inflammatory pain model [25]. Nagy et al. showed that some GluR1 subunits in the dorsal horn were phosphorylated at Serine ^845 ^site following noxious stimulation [15]. Since phosphorylation at this site is necessary for the insertion of GluR1-containing receptors, this provides the further evidence that noxious stimulation may induce insertion of GluR1-containing receptors in spinal neurons. The signaling pathways that drive the insertion of GluR1 subunits into the plasma membrane during LTP *in vitro *also require the activity of neuronal CaMKII [26]. In spinal neurons, intra-thecal application of a CaMKII inhibitor, KN-93, before the painful visceral stimulus, apparently inhibits the GluR1 accumulation in the plasma membrane fraction [24]. This suggests that the painful visceral stimulus promote synaptic delivery of GluR1-containing receptors in a CaMKII-dependent manner. Larsson et al. showed that in a rat model of acute inflammatory pain, hyperalgesia was associated with an elevated density of GluR1-containing AMPA receptors as well as an increased ratio of GluR1 to GluR2/3 subunits at synapses. It suggests a significant membrane translocation of GluR1-containing AMPA receptors to a spinal nociceptive synapse during acute noxious stimulation [27]. In an animal model of complete Freund's adjuvant (CFA)-induced persistent inflammatory pain, Park et al. found that CFA-induced inflammation did not change the total expression or distribution of AMPA receptor subunits, GluR1 and GluR2 in spinal dorsal horn, but did alter their subcellular distribution. They demonstrated that the amount of GluR2 was markedly increased in the crude cytosolic fraction and decreased in the crude membrane fraction from the ipsilateral L4-5 dorsal horn at 24 hour post-CFA injection. Conversely, the level of GluR1 was significantly reduced in the crude cytosolic fraction and increased in the crude membrane fraction from the ipsilateral L4-5 dorsal horn at 24 hour post-CFA injection [5]. All the data demonstrated that the regulation of the spinal AMPA receptors in postsynaptic membranes through the receptor trafficking may play a remarkable role in the AMPA receptor-mediated nociception.

## Functional regulation of spinal AMPA receptors through phosphorylation of receptor subunits

**Figure 2:**
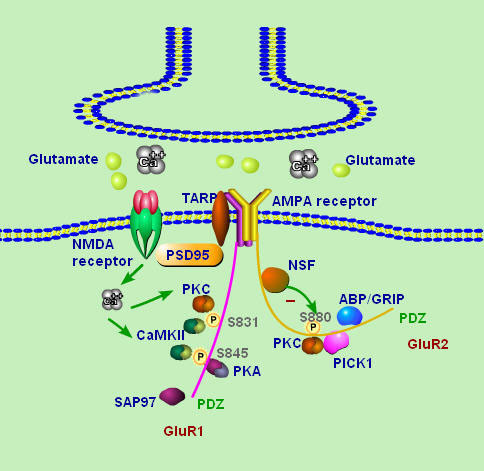
**The phosphorylation of AMPA receptor subunits and its interaction with partner proteins in spinal neurons**. In spinal neurons, it has shown that PKA mediates the phosphorylation of serine at the Serine^845 ^site, and PKC targets the Serine^831 ^site following noxious stimulation. More specifically, CaMKII mediates the phosphorylation of GluR1 subunit of AMPA receptor at both Serine^831 ^and Serine^845 ^sites in spinal neurons after strong noxious peripheral stimulation. GluR2 subunits of AMPA receptors may bind to GRIP and PICK1, which play an important role in the GluR2 trafficking. GRIP anchors GluR2 subunits at synapses, whereas PICK1 brings PKC to synaptic GluR2. PKC phosphorylates GluR2 Serine^880 ^to release GluR2 from GRIP and to promote GluR2 internalization. CFA-induced peripheral inflammation could induce GluR2 internalization in dorsal horn neurons. Stargazin binds to GluR1, 2, and 4. Binding of the Stargazin C-terminal tail to PSD-95 mediates the synaptic targeting of surface AMPA receptors. SAP 97 binds to the GluR1 C-terminus, interacts with the actin-associated protein 4.1N and is implicated in GluR1 synaptic insertion. NSF protein plays a role in membrane fusion processes and also interacts with GluR2 and GluR3 subunits of AMPA receptors. The following abbreviations are used: PKA, protein kinase A; PKC, protein kinase C; CaMKII, Ca^2+^/calmodulin-dependent protein kinase II; GRIP, glutamate receptor interacting protein; PICK1, protein interacting with C-Kinase; CFA, complete Freund Adjuvant; TARP, transmembrane AMPA receptor regulatory proteins; PDZ, postsynaptic density zone; PSD, postsynaptic density; SAP 97, synapse-associated protein 97; NSF, N-ethylmaleimide sensitive fusion.

A variety of extra-, intra-cellular signals following peripheral noxious stimulation trigger cellular and molecular changes at transcriptional, translational, or post-translational levels, and theses events may contribute to the central sensitization. The phosphorylation of membrane receptors is an important post-translational mechanism underlying synaptic plasticity in nervous systems as well as in pain modulation. Strong noxious stimulation in the periphery tissues may activate several protein kinase cascades, such as CaMKII, PKA, PKC, and PKG, which play an important role in the phosphorylation of glutamate receptors in spinal nociceptive neurons [23,28-30]. The increased sensitivity of glutamate receptors through the phosphorylation regulated by multiple intra-cellular protein kinases may contribute to the enhanced responsiveness of dorsal horn neurons during central sensitization [31-33]. As an important class of glutamate receptors, phosphorylation of AMPA receptor subunits has been widely investigated in relation to processes of synaptic plasticity in different brain regions. It has been demonstrated that phosphorylation of AMPA receptor subunits may potentiate their activity, influence their interaction with intracellular partner proteins, and promote their expression at the plasma membrane during synaptic plasticity [34-37]. All these intracellular events triggered by phosphorylation of AMPA receptor subunits may contribute to the enhanced efficacy of glutamatergic synapses (Figure 2). In spinal neurons, accumulating evidence supports the important role of the regulation of AMPA receptors by phosphorylation in spinal nociceptive process.

The intracellular C-terminal domains of AMPA receptor subunits may allow subunit-specific regulation by phosphorylation. There are several protein phosphorylation sites located on the C-terminal region, which are working targets of PKA, PKC, and CaMKII. *In vitro *studies on hippocampal slices show that AMPA receptors can be directly phosphorylated on at least 12 distinct phosphorylation sites. Site-directed mutagenesis and phosphopeptide analysis has identified the two major phosphorylation sites on GluR1: Serine^845^, which is phosporylated majorly by PKA, and Serine ^831^, which is phosphorylated majorly by PKC [38]. The phosphorylation of Serine^845 ^in GluR1 by PKA regulates the open-channel probability of AMPA receptors; whereas the phosphorylation of Serine^831 ^by PKC changes channel conductance [39]. CaMKII was also found to phosphorylate Serine^831 ^in GluR1 and contributes to the single-channel conductance of the receptor and may increase AMPA receptor conductance during LTP [35,40]. In spinal neurons, our group has shown that PKA mediates the phosphorylation of serine at the Serine^845 ^site, and PKC targets the Serine^831 ^site following noxious stimulation [23,31,41]. Furthermore, we have demonstrated that AMPA receptors showed enhanced responsiveness to nociceptive stimulation through this phosphorylation processing during central sensitization. More specifically, CaMKII may impact the phosphorylation of GluR1 subunit of AMPA receptor at both Serine^831 ^and Serine^845 ^sites in neurons in the spinal cord after strong noxious peripheral stimulation [23]. Phosphorylation of GluR1 at Serine^831 ^by CaMKII in central sensitization is consistent with the results of studies of LTP in the hippocampus. CaMKII inhibitor, KN-93, partially blocked the phosphorylation of GluR1 at the Serine^845 ^site, which is a PKA phosphorylation site either. CaMKII may indirectly mediate the phosphorylation of GluR1 at the Serine^845 ^site through adenylate cyclase and PKA, since the Ca^2+^-calmodulin complex can stimulate adenylate cyclase, and subsequently activate more cAMP production and PKA activity. Lu et al. demonstrated that phosphorylated GluR1 (phosphorylated GluR1-Serine^845 ^and phosphorylated GluR1-Serine^831^) might play a role in the induction of inflammatory pain but not neuropathic pain [42].

The phosphorylation of GluR2 plays an important role in the receptor clusters during synaptic plasticity and persistent pain. It has been demonstrated that GluR2 may be phosphorylated on Serine^880 ^by PKC in *in vitro *and in transfected cells [36]. AMPA receptor GluR2 subunit may bind to cellular partner proteins, such as glutamate receptor interacting protein (GRIP) and this signal protein interacting with C-Kinase (PICK1), which plays an important role in the synaptic GluR2 trafficking [43,44]. As the PDZ domain-containing proteins, GRIP anchors GluR2 at synapses while PICK1 brings PKC to synaptic GluR2. PKC phosphorylates GluR2 at Serine^880 ^to release GluR2 from GRIP and to promote the internalization of GluR2 [36]. The interference of the interaction between GluR2 and GRIP by GluR2 phosphorylation apparently disrupts AMPA receptor GluR2 clusters [45]. It has been demonstrated that complete Freund's adjuvant (CFA)-induced peripheral inflammation may induce synaptic GluR2 internalization in spinal dorsal horn neurons and this internalization was initiated by PKC-mediated GluR2 phosphorylation at Serine^880^. Subsequently, the disruption of GluR2 binding to its synaptic anchoring protein (GRIP) can result in a switch of GluR2-containing AMPA receptors to GluR2-lacking AMPA receptors. This disassociation may also increase AMPA receptor Ca^2+ ^permeability at the synapses in dorsal horn neurons [46]. Moreover, preventing CFA-induced spinal GluR2 internalization through targeted mutation of the GluR2 PKC phosphorylation site could reduce CFA-evoked hypersensitivity during the maintenance of nociceptive process. It may suggest a potential strategy in developing selective blockers targeting receptor single subunit or specific post-translational points.

The phosphorylation of another subunit, GluR4 has also been demonstrated to play an important role in spinal nociception. GluR4 is the most rapidly desensitizing subunit of AMPA receptors and is phosphorylated at Serine^842^, within its C-terminal domain [47]. PKA, PKC, and CaMKII may phosphorylate at Serine^842 ^site of GluR4 very well. Threonine^830 ^is also found as an important phosphorylation site on GluR4 by PKC [47]. Recently, Polgar et al. reported that postsynaptic GluR4-containing AMPA receptors were involved in spinal nociceptive transmission [48]. However, how GluR4 phosphorylation contributes to spinal nociception needs further investigation.

## Regulation of the interactions between AMPA receptor subunits and associated partner proteins in spinal cord neurons during nociception

In the past decades, it has been demonstrated that a number of proteins interact with the intracellular C-termini of postsynaptic AMPA receptor GluR1-4 subunits. These proteins are closely associated with the trafficking of AMPA receptor subunits and the subsequent regulation of intracellular signal transduction cascades. It has been demonstrated from previous studies that the regulated synaptic insertion of AMPA receptor subunits through the interactions of subunits with protein partners can play a critical role in spinal dorsal horn sensitization [46,49] (Figure 2).

Other critical PDZ-domain-containing proteins, such as GRIP and ABP were found to interact with the xS/TxV motif at the extreme C-terminal of short forms of AMPA receptor subunits [43,50]. The interaction of GluR2 and GRIP plays a significant role in clustering AMPA receptors at spinal excitatory synapses in a model of neuropathic pain [13]. It has been found that phosphorylation of GluR2 at Serine^880 ^by PKC reduces the affinity of GluR2 for GRIP, thus, released GluR2 from GRIP and promotes the internalization of GluR2 [36]. Recently, the internalization of spinal GluR2 subunit of AMPA receptor has also been demonstrated in an animal model of persistent inflammatory pain [46]. The internalization of phosphorylated GluR2 subunit of AMPA receptors may result in an increased synaptic ratio of GluR1 to GluR2 subunit since the membrane translocation of phosporylated GluR1 subunit to a spinal nociceptive synapse may occur during noxious stimulation [24,27]. The increased synaptic ratio of GluR1 to GluR2 subunit, in turn, might result in an increase in spinal Ca^2+^-permeable AMPA receptors in the spinal dorsal horn. A study in GluR2-deficient mice has shown that an increase in spinal Ca^2+^-permeable AMPA receptors might facilitate nociceptive plasticity and enhance long-lasting inflammatory hyperalgesia [51]. Taken together, the interaction between GluR2 and GRIP in spinal neurons might be involved in the nociceptive regulation through affecting the internalization of GluR2.

PICK1 has also been found to bind to the C-terminal of GluR2, -3, -4 subunits of AMPA receptors through the PDZ domain [44]. PICK1 was observed to participate in regulating synaptic AMPA receptor at various levels [52]. On one hand, PICK1 can dimerise and the dimers may induce the aggregation of AMPA receptors in heterologous expression systems [44]. On the other hand, PICK1 dimers could bring PKCα to AMPA receptors and present a selective phosphorylation site of GluR2 at Serine^880 ^[53-55]. This phosphorylation of GluR2 on Serine^880 ^may further impede the affinity of GluR2 for GRIP, release GluR2 from GRIP-GluR2 complex and finally lead to the internalization of GluR2 subunits.

As a family member of trans-membrane AMPA receptor regulatory proteins (TARP), Stargazin binds to GluR1, -2, and -4 at sites other than PDZ target motifs. Stargazin plays a critical role in the regulation of AMPA receptor trafficking between synaptic and extra-synaptic sites. Binding of the C-terminal tail of Stargazin to PDZ domains of a number of synaptic scaffolding proteins, such as PSD-95 (an interacting protein associated with NR2 subunits of the NMDA receptors) may mediate the synaptic targeting of surface AMPA receptors [56]. PSD-95 has been shown to play a crucial role in spinal mechanisms of central sensitization following noxious stimuli [57-59]. Thus, it may suggest that the Stargazin-mediated interaction of AMPA receptors with PSD-95 is possibly implicated in the spinal sensitization. The translocation of AMPA receptors from the cytosol to the plasma membrane also requires the involvement of Stargazin. It has been showed that an over-expression of Stargazin increases the number of extra-synaptic AMPA receptors without affecting AMPA receptor-mediated synaptic transmission. But, the over-expression of PSD-95 may enhance AMPA receptor-mediated synaptic responses. Studies may suggest a critical role of Stargazin in the regulation of AMPA receptor trafficking from extra-synaptic to synaptic sites, thus it leads to the controllable events of AMPA receptor-PSD-95 complex [56].

The GluR1 subunit of AMPA receptors may also have the interaction with some regulatory proteins through its termini. In hippocampal neurons, the interaction of GluR1 with its partner proteins has been thought to be involved in controlling receptor trafficking and synaptic insertion during neuronal plasticity [52,60,61]. It has been studied that the CaMKII-dependent trafficking of GluR1 subunit to dendritic spines required the PDZ target motif in GluR1 subunit [62]. Another PDZ domain protein, synapse-associated protein 97 (SAP 97) binds to the C-terminus of GluR1 subunit and interacts with the actin-associated protein 4.1N (which can also bind GluR1 subunit). Their interaction was reported to be involved in the synaptic insertion of GluR1 subunit [63]. Since more evidences support that the role of phosphorylation of GluR1 subunit of AMPA receptors in acute and chronic inflammatory pain [42], it may propose a novel mechanism that the interaction of GluR1 subunit of AMPA receptors with its partner proteins was implicated during spinal hyper-sensitivity.

The role of Non-PDZ domain-containing protein, such as N-ethylmaleimide sensitive fusion (NSF) protein (an ATPase), in neuronal membrane fusion processes and regulatory interaction with GluR2 and GluR3 subunits of AMPA receptors has been investigated. The interaction may be responsible for the insertion and stabilization of AMPA receptors containing GluR2/3 subunits [64]. Garry et al. reported that cell-permeable blocking peptides targeting for interactions of GluR2 with NSF or for GluR2/3-GRIP/PICK1 complex may have anti-hyperalgesic effects in a neuropathic pain model [13]. Recently, Katano's group had also shown that NSF is involved in central sensitization in the spinal cord through a switch of GluR2 subunit composition in a CFA-induced peripheral inflammatory pain model [65].

In summary, the interaction of AMPA receptor subunits with their partner proteins was extensively involved in the processes of the regulation of post-translational modification, such as trafficking, internalization and surface expression. All these events may influence the AMPA receptor-mediated synaptic transmission as well as spinal central sensitization.

## Functional regulation of spinal AMPA receptors through the composition switch of its subunits

Incorporation of GluR2 subunits into heteromeric AMPA receptor strongly reduces the permeability of the AMPA receptor channel to Ca^2+ ^ion influx [66]. Thus, the switch in the composition of synaptic AMPA receptors may greatly influence the AMPA receptor-mediated synaptic efficacy. The rapid alterations in the composition of synaptic AMPA receptors induced by various stimuli may occur in the cerebellum, the hippocampus and the cortex. Current evidence also shows that a persistent activation of primary nociceptive afferent fibers may rapidly regulate synaptic AMPA receptor composition in the spinal neurons. For example, noxious stimuli can change the expression ratio of synaptic AMPA receptor subunits GluR1 to GluR2 in a visceral pain model [24]. Peripheral injury may also alter the spinal AMPA receptor composition in inflammatory pain process [65]. Hartmann et al. reported that the GluR1 and GluR2 subunits reciprocally modulate spinal synaptic plasticity and inflammatory pain in GluR1-knockout mice [51]. A reduction in the number of Ca^2+^-permeable AMPA receptors and density of AMPA-channel currents in spinal neurons in GluR1-deficient mice is accompanied by a loss of nociceptive plasticity and a reduction in acute inflammatory hyperalgesia [51]. In contrast, an increase in spinal Ca^2+^-permeable AMPA receptors in GluR2-deficient mice facilitated nociceptive plasticity and enhanced long-lasting inflammatory hyperalgesia [51]. It supports that peripheral inflammation might induce the switch of Ca^2+^-permeable AMPA receptors in dorsal horn neurons [46,65,67].

The rapid alterations in the composition of synaptic AMPA receptors induced by physiologic activity or noxious stimuli can be achieved by modulating the phosphorylation status of GluR1 and GluR2 subunits and their binding to PDZ domain-containing synaptic scaffolding proteins. Thus it may change the membrane targeting and synaptic availability of AMPA receptors. CFA-induced peripheral inflammation could induce synaptic GluR2 internalization in spinal dorsal horn neurons during nociception processing and this internalization might be initiated by the phosphorylation of GluR2 at Serine^880 ^by PKC. It subsequently disrupted the binding of GluR2 subunits to synaptic anchoring protein (GRIP) and result in a switch of GluR2-containing AMPA receptors to GluR2-lacking AMPA receptors. Finally, an increased AMPA receptor Ca^2+ ^permeability induced more Ca^2+ ^influx in dorsal horn neurons [46]. NSF is also reported to be involved in central sensitization in the spinal cord through a composition switch of GluR2 subunit following CFA-induced peripheral inflammation [65]. Additionally, potential changes in GluR2 mRNA editing in disease states may modulate nociceptive responses by altering AMPA receptor composition in the spinal dorsal horn. Dysfunctional GluR2 editing has been reported in the human spinal cord in several neurodegenerative diseases, such as amyotrophic lateral sclerosis. It deserves to be investigated to observe whether similar changes occur in patients suffering from chronic inflammatory or neuropathic pain [68].

## Concluding remark

AMPA receptor subunits may present unique characteristics in the receptor trafficking, subunit phosphorylation, interaction with partner proteins, and composition changes in response to noxious stimuli. All of these molecular events are closely associated with the development or maintenance of persistent pain. The phosphorylation of AMPA receptor subunit may be the most important process because it can trigger or affect other associated processes. For example, the phosphorylation of GluR1 subunit through CaMKII drives synaptic delivery of GluR1-containing receptors following painful stimuli. The phosphorylation of GluR2 subunit at Serine^880 ^by PKC may lead to disrupt the interaction between GluR2 and GRIP and follow by GluR2 internalization. The phosphorylation status of GluR1 and GluR2 subunits modulates the rapid alterations in the composition of synaptic AMPA receptors. The development of selective drugs based on targeting receptor single subunit- or specific post-translational site may provide an effective novel strategy in treating persistent or chronic pain.

## Competing interests

The authors declare that they have no competing interests.

## Authors' contributions

YW participated in the design of the review and drafted the manuscript. JW, ZW and QL assisted with the preparation of the manuscript and figures. YY and LF conceived of the review and participated in its design and helped to draft the manuscript. All authors read and approved the final manuscript.

All authors have read and approved the final manuscript.
